# CK8/18 and Claudin Profile of Female Breast Cancers from Botswana

**DOI:** 10.1177/11782234261471677

**Published:** 2026-08-27

**Authors:** Andrew Khulekani Ndlovu, Ishmael Kasvosve, Richard Naidoo, Dhiren Govender

**Affiliations:** 1 Department of Medical Laboratory Science, Faculty of Medicine and Health Sciences, 54547 University of Botswana, Gaborone, Botswana; 2 Division of Anatomical Pathology Faculty of Health Sciences, University of Cape Town, Cape Town, Republic of South Africa

**Keywords:** female breast cancer, IHC, CK8/18, claudin-3, claudin-4, claudin-7, claudin-low

## Abstract

**Background:**

Globally, breast cancer mortality is rising particularly in transitioning countries where it is exacerbated by late diagnosis and poor diagnostic infrastructure. The dearth of data on the biology of breast tumours from under-studied populations compromise management of breast cancer patients resulting in poor patient outcomes. We conducted a study to determine the prevalence and impact of Claudin-low and CK8/18 in the Botswana female breast cancer cohort.

**Design:**

This laboratory experimentally retrospective cross-sectional study was carried out on archived formalin-fixed paraffin embedded (FFPE) tissue obtained from mastectomy specimens.

**Objectives:**

To determine the protein expression of CK8/18 and Claudin-3, Claudin-4 and Claudin-7 on breast mastectomies from Botswana female breast cancer cohort.

**Methods:**

CK8/18, Claudin 3, Claudin 4 and Claudin 7 were Immunohistochemical determined on 125 previously molecular classified Botswana female mastectomies. Statistical software STATA and SPSS were used to determine the relationship between CK8/18 & Claudin expression, clinicopathological variables, breast cancer molecular subtypes, CK5/6 and EGFR.

**Results:**

The prevalence of Claudin-low tumours in our cohort was 22% (27/125) had a significant relationship with histological subtype, p=0.002. Claudin 3 and Claudin 4 were highly expressed in 54% (67/125) and 53% (66/125) of breast tumours respectively. Claudin 3 and claudin 4 significantly correlated with molecular breast cancer subtypes, p= 0.001. Claudin 7 was expressed in 47% (59/125) of all breast tumours and had a significant relationship with CK5/6, p=0.029. CK8/18 was highly expressed by 64% (80/125) of mastectomies and correlates with; Tumour grade (p= 0.005) and breast cancer molecular subtypes (p=0.002).

**Conclusion:**

Diagnostic stratification of breast tumours should include Claudin-low tumours as they respond differently to therapy when compared to the intrinsic breast cancer molecular subtypes. There is also a significant CK8/18 signaling that warrants deployment of anti-CK8/18 antagonists where ER is poorly expressed.

## Introduction

In 2020, globally 2.3 million women were diagnosed with breast cancer and 685 000 succumbed to the disease. Nearly 7.8 million women were diagnosed with breast cancer in the past 5 years, thus making it the world’s most prevalent cancer.^1^ Seventy-four-thousand and seventy-two, (74 072) cancer associated deaths in Africa were due to breast cancer. One hundred and sixty-eight thousand, six hundred and ninety (168 690) cases were estimated to have occurred in 2018. The age-standardized incidence rate stood at 37.9/100 000 in Africa, varying from 6.9/100 000 in the Gambia to 69.6/100 000 in Mauritius. According to Freihat et al, Africa experienced a mortality to incidence rate of 0.510 in 2022 and breast cancer incidence is projected to reach 1.118 million by 2050.^2^ One the plausible reason given is that there is lack of early diagnosis and treatment infrastructure.^2-4^

Breast cancer is one of the most commonly diagnosed cancers among women in sub-Saharan Africa. In Botswana, breast cancer represents the second most common cancer diagnosed among women, after cervical cancer.^1^ There is evidence that women of African descent present with large, clinically advanced stage tumours across all molecular subtypes.^5-12^

Claudins are tight junctions which control movement of substances (including growth factors), constraining and repressing tumours.^13,14^ Tumours that have low expression of claudins proteins are termed Claudin-low. The claudin-low breast cancer subtype is the newest group to be identified by gene expression profiling studies^15,16^ and is characterized by low expression of genes involved in the formation of tight junctions and intercellular adhesions. Key among them being claudin-3, -4 and -7. The claudin-low subtype shares the low expression of HER2 and luminal gene cluster of the basal-like subtype. The claudin-low subtype overexpresses genes related to immune response and as a result shows prominent inflammatory cell infiltration.^16,17^ They have a poor prognosis. Furthermore, they express a subset of genes related to mesenchymal differentiation and epithelial mesenchymal transition.^18,19^

Globally, the overall prevalence of Claudin-low is between 12-14% and 25-39% of claudin-low tumours are found within the triple-negative breast cancer (TNBC) molecular subtype.^20^ Genotyping studies have estimated hormonal receptor positive claudin-low subtype to be between 15 and 25%.^18^ They correspond to infiltrating ductal carcinomas special type which present with metaplastic or medullary morphology, have a poor long-term prognosis and their response to chemotherapy is in between basal-like and luminal subtypes.^17^

Cytokeratins (CKs) are “epithelial cell specific” intermediate filaments expressed in epithelial cells of many organs including the breast.^21^ CK18 is found in luminal epithelia while basic or neutral cytokeratins are present in the myoepithelial component of the mammary gland.^21^ CK8/18 provides the intracellular scaffold for support and maintenance of cell integrity^22,23^ CK18 has been found to participate in cellular processes such as apoptosis, mitosis and signaling through various pathways, including the Wnt pathway.^22^

CK8/18 has been found to aid treatment in breast cancer by preventing oestrogen signaling associated cellular proliferation.^24,25^ It is used as a diagnostic marker to separate luminal breast cancer subtypes from the basal subtype. It is also used as a prognostic marker that predicts good response to oestrogen antagonists in breast tumours which co-express ER.^21,26^ A high CK8/18 expression is associated with sporadic breast cancer and corresponds to good prognosis,^27,28^ while it is poorly expressed in *BRCA* associated breast tumours^29^). CK8/18 may be used to distinguish between the special histological types of breast cancer; it is highly expressed in mucinous, ILC and tubular carcinoma as opposed to adenoid cystic, medullary and metaplastic carcinoma.^30^ Gene expression profiling (GEP), in breast cancer has also confirmed CK8/18 as a marker for luminal A breast tumours and subsequently it has since been used to differentiate luminal A tumours from other molecular subtypes.^31-33^

To understand the biology behind the large sizes of breast tumours in the Botswana cohort,^34^ we studied CK8/18 (an integral part of the cell cytoskeleton which maintains cellular integrity and is involved in both cell division and apoptosis) and the 3 claudin proteins (claudin-3, -4 and -7) that are an integral part of tight junctions which are tasked with controlling movement of substances (including growth factors), and additional acts as a barrier and constrainer of tumour growth.^13,14,35^

## Methods

### Study Design, Study Site and Sample Size

This retrospective cross-sectional study was carried out on archived formalin-fixed paraffin embedded (FFPE) tissue obtained from mastectomy specimens received between August 1^sts^ 2006 and December 31^st^, 2009. The study followed reporting guidelines for evidence based-statistical analysis and methods in biomedical research (SAMBER) checklist for laboratory-based experiments,^36^ see completed checklist Supplementary file S4. The study samples were collected from National Health Laboratory, Gaborone Botswana and from Nyangabwe Referrals hospital Pathology Department, City of Francistown Botswana. Immunohistochemistry analysis was conducted in the Division of Anatomical Pathology Research Laboratory, University of Cape Town, South Africa.

### Ethical Considerations

Ethical approval was obtained from the University of Botswana Office of Research and Development Institutional Review Board (RES/IRB/1380). A research permit was obtained from the Botswana Ministry of Health Research Division (PPME 13/18/1) and ethical clearance approvals were obtained from Princess Marina (PMH 5/75 &79) and Nyangabgwe Hospital Research and Ethics Committees (NRH/1/2). Permission to transport residual FFPE tissue samples to South Africa to conduct research was granted by the Ministry of Health MH 3/87 III. Further ethical approval was also granted by the University of Cape Town, Faculty of Health Sciences, Human Research Ethics Committee (HREC 620/2013). The archived tumour blocks were de-identified before enrolment into the study in keeping with the provisions of ISO 15189.

### Study Samples

**Figure 1.**
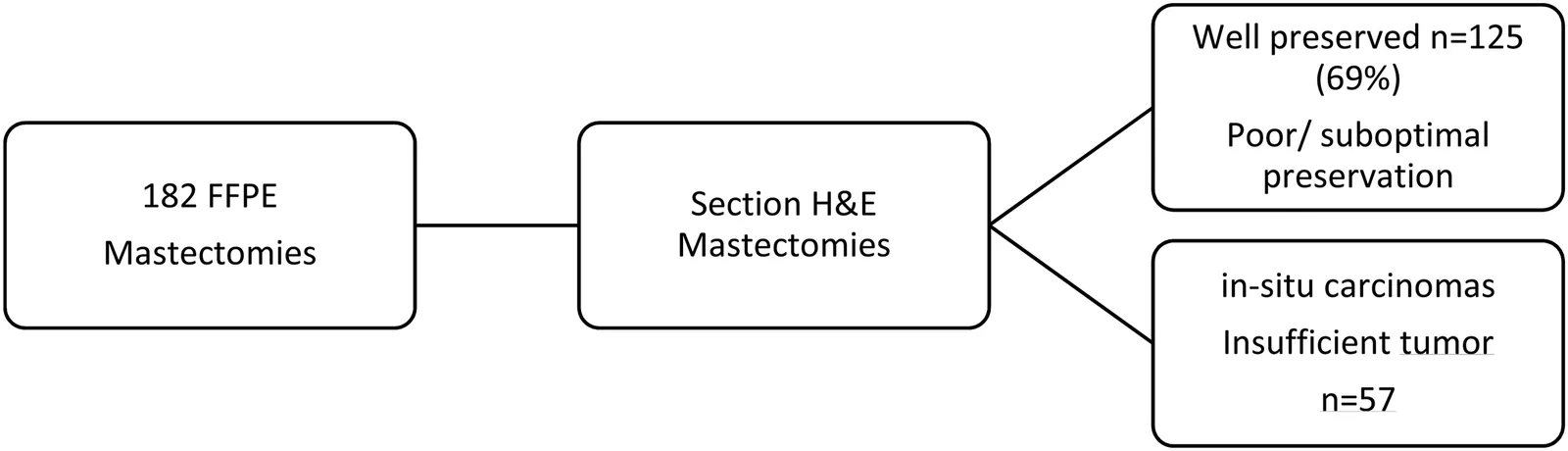
Cases enrolment flow chart

In total 182 mastectomy specimens were obtained from the two Botswana anatomical pathology laboratories as previously described^34^ and the cases were reviewed and classified according to WHO histological classification by a second pathologist at University of Cape Town and 125 (69%) were confirmed as female breast cancer Figure 1. The remaining cases were either in-situ carcinomas or had insufficient tumour or had suboptimal fixation and were excluded from further analysis.

### Laboratory Investigations

Selected breast cancer and positive control FFPE tissue blocks were sectioned at 3µm, using a Leica RM2125 RTS microtome. The sections were floated on a water bath and picked up on HistoBond adhesive glass slides (Paul Marienfeld GmbH & Co. KG, Germany). The sections were then dewaxed for 2 minutes in three changes of xylene, cleared in absolute alcohol and followed by washing under running tap for 5 minutes. Antigen retrieval was performed as shown in Table 1 in a Russell Hobbs RHPC 6L 4110 pressure cooker. Immunohistochemistry was performed as per appended protocol appendix 1 for CK8/18, Claudin-3, Claudin-4 and Claudin-7.

**Table 1.** Antibodies and Immunohistochemical Staining Conditions

Antibody	Clone	Control	Retrieval buffer	Dilution	Incubation time at RT
CK 8/18	NCL-L-5D3	skin	Citrate Buffer pH 6	1:50	60 mins
Claudin-3	rabbit polyclonal	Small intestine	Citrate buffer pH 6	1:200	60 mins
Claudin-4	rabbit polyclonal	tonsil	Citrate buffer pH 6	1:100	60 mins
Claudin-7	rabbit polyclonal	Human colon cancer	Citrate buffer pH 6	1:200	60 mins

### Immunohistochemistry Scoring

• An H-scoring system was used to determine the IHC expression of CK8/18. This scoring system was used as it is a semi-quantitative method able to account for tumour heterogeneity.^37,38^ Cytoplasmic expression of CK8/18 was analysed by evaluating the intensity and corresponding proportion of cells staining in a tumour. Intensity was graded as follows: 0 (negative), weak (1), moderate (2) and strong (3). The H-score was the sum score generated by multiplying each intensity of staining cells by the corresponding proportion of tumour cells staining Σ [ (I_o*_P_0_) + (I_1_*P_1_) _+_ (I_2*_P_2_) + (I_3_*P_3_)]. For heterogeneous tumours, the score was generated by taking the sum of scores for each intensity. The expression-score ranged from 0-300. An expanded immune reactivity score system (IRS) was used for claudin expression from 0-9 to 0-12^37^. IRS was assessed using a combination of proportion and intensity of cell staining. Scores of percentage staining were assigned a scale of 0-4 where, negative = 0; 1-10% = 1; 11-25% = 2; 25-50 = 3 and > 50% = 4 and the intensity scored 1 to 3 as described above. The two scores were multiplied to give an overall score of either 0, 1, 2, 3, 4, 6, 8, 9 and 12 of which 0 was recorded as negative, 1-3 was considered weak positive, 4 and 6 were considered moderate positive and 8, 9 and 12 were considered strong positive. For heterogeneous tumours, the score was generated by taking the sum of scores for each intensity. Score of 0-3 were interpreted as claudin-low, while scores of 4-12 were interpreted as claudin-high.

### Classification of breast Cancer From Botswana (2006-2009)

The 125 female breast cancer tissues enrolled in the study were previously reviewed and classified by one pathologist following the WHO histological classification^39^ their 4-IHC immunohistochemistry profile, EGFR and CK5/6 expression determined as shown in Table 2 below.

**Table 2.** Clinicopathological Data of 125 Enrolled Breast Cancer Cases

Variable		n (%)
Age	**≤50 years**	45 (36)
	**>50 years**	80 (64)
Clinicopathological profile	**Tumour Grade**	
	I	38 (30)
	II	50 (40)
	III	29 (24)
	*Mucinous	8 (6)
	**Tumour size**	
	<2 cm (AJCC *Tmi to T1c*)	6 (5)
	2-5 cm (AJCC *T2*)	57 (46)
	>5 cm (AJCC *T3 & T4*)	52 (41)
	Unknown	10 (8)
	**Lymph node involvement** **	
	Yes, n (%)	83 (66.4)
	No, n (%)	28 (22.4)
	Unknown	14 (11.2)
**Histopathology profile**		
Histology Type	Invasive ductal-NOS, n (%)	93 (74)
	Special type, n (%)	30 (24)
	Invasive lobular, n (%)	2 (2)
**Immunohistochemistry**		
CK5/6	Positive	16 (13)
	Negative	109 (87)
EGFR	Positive	42 (34)
	Negative	83 (66)
**Molecular subtypes** **		
​	Luminal A	44 (35)
	Luminal B	23 (18)
	Luminal B HER2 Positive	9 (7)
	Triple negative	32 (26)
	HER2 enriched	17 (14)

*Mucinous carcinoma cases were not allocated grades (8 cases).

**Due to lack of accuracy of data on type lymph node, involvement refers to overall lymph node involvement rather than type of lymph node.

### Statistical Analysis

Stata software, Version 15.1, StataCorp, Texas USA was used to conduct statistical analysis. Categorical data was summarised using frequencies. Bivariate analysis to test for association between Claudins (3,4,7 and Claudin low) expression and clinicopathological variables (age at diagnosis, tumour size, lymph node involvement, histological type and grade). Furthermore, bivariate analysis between Claudins (3,4,7 and Claudin low) expression and breast cancer molecular subtypes (luminal A, Luminal B, Luminal B Her2 positive, HER2 Enriched and TNBC) & biomarkers (CK5/6, EGFR) was conducted using the Chi-square test at 95% confidence interval. However, when the assumptions for the chi-square test were not met, a Fisher`s exact test was used for the analysis. Results were considered statistically significant when p-value was <0.05. The results were presented in tables. For Multivariate logistic regression only variable with a p-value ≤ 0.2 were included in the final model and results were statically significant if p value <0.05 at 95% confidence interval.

## Results

### CK8/18 Expression

**Figure 2.**
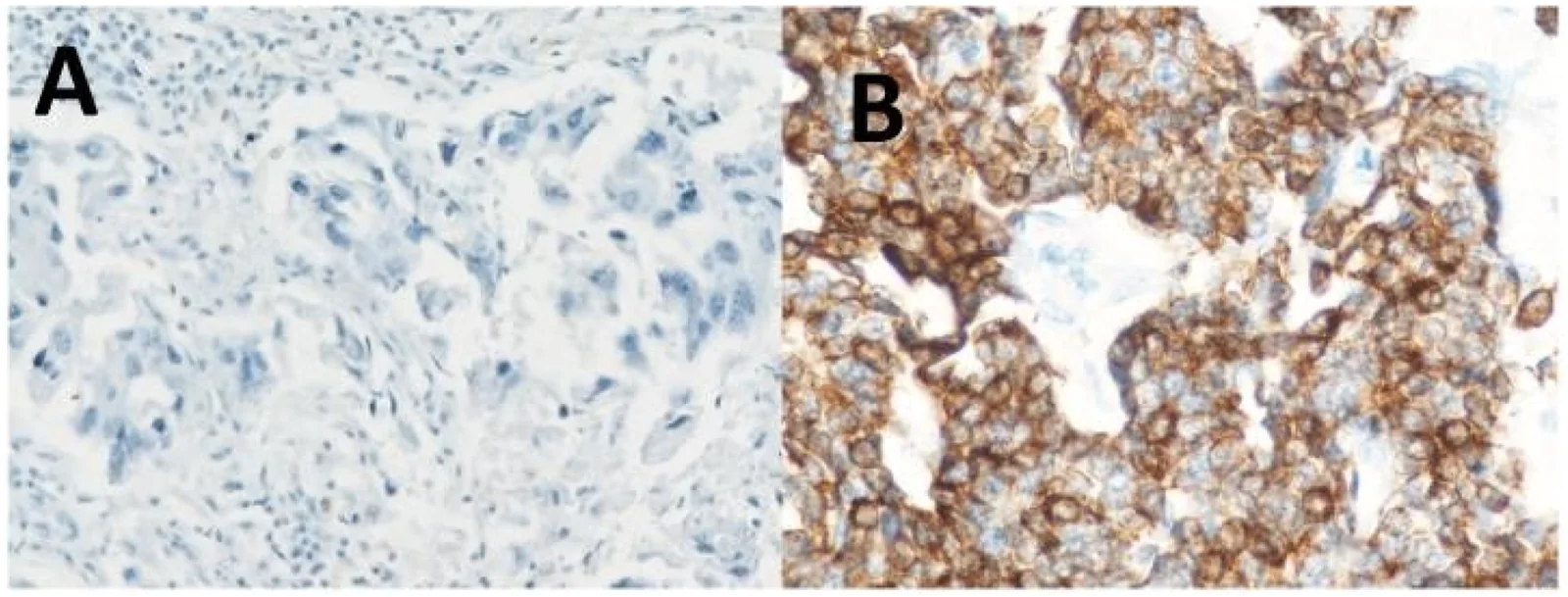
Showing CK8/18 expression H scores (A=0 and B=280)

CK8/18 was expressed in varying degrees by 113/125 (90%) of tumours. H_score_ = Σ [ (I_o*_P_0_) + (I_1_*P_1_) _+_ (I_2*_P_2_) + (I_3_*P_3_)] The mean H score was 125 (range 0-300, median score 117). Sixty-four percent (80/125) of tumours had an H score of 100 or more CK8/18, 26% (33/125) expressed between 99 and 30 and 10% (12/125) of tumours did not express CK8/18, Figure 2.

CK8/18 was inversely correlated with tumour grade, Table 3. CK8/18 expression decreased with an increase in grade as in table 3 below. CK8/18 expressing breast tumours were well differentiated; grade 1 tumours had an average H-score of 165 (median 180, range 0-300) compared to Grade 2 tumours with an average H-score of 133 (median 120, range 0-300). Grade 3 tumours had an average expression of 75 (median 70, range 0-100), their CK8/18 expression was as disrupted as in mucinous tumours which had an average expression of 64 (median 70, range 0-100). Twenty-five percent of mucinous (2/8) mucinous tumours expressed CK8/18.

**Table 3.** Correlation of CK8/18 With Grade and Molecular Subtypes

Variable	CK8/18			P Value
Tumour grade, n=117*				0.005
​	**Positive, n (%)**	**Negative, n (%)**	**Average H**_**score**_	​
Grade 1, n = 38	29 (76)	9 (24)	165	​
Grade 2, n = 50	37 (74)	13 (26)	133	​
Grade 3, n = 29	12 (41)	17 (59)	75	​
*Mucinous, n=8	2 (25)	6 (75)	64	​
**Molecular subtypes, n=125**				0.002
​	**Positive, n (%)**	**Negative, n (%)**	**Average H**_**score**_	​
Luminal A, n = 44	34 (77)	10 (23)	164	​
Luminal B, n = 23	15 (65)	8 (35)	124	​
Luminal B HER2E, n = 9	9 (100)	0 (0)	124	​
HER2 Enriched, n = 17	8 (47)	9 (53)	101	​
TNBC, n = 32	14 (44)	18 (56)	78	​

*8 cases were mucinous.

Furthermore, CK8/18 had a significant correlation with molecular subtype p=002, 73 % (58/80) of CK8/18 positive tumours were of Luminal subtypes. Conversely, 60% (27/45) of CK8/18 negative tumours were HER2 Enriched and TNBC subtypes.

For a multivariate analysis molecular subtype (p=0.002) and tumour grade (p=0.004) were associated with CK8/18 expression.

For Bivariate analysis there was no relationship between CK8/18 expression and age (p=0.847), tumour size (p=0.326), histological type (p=0.165) and lymph node involvement (p=0.269). CK8/18 expression did not correlate with biomarkers; CK5/6 expression (p=0.785) and EGFR expression (p=0.075) See Table S1 in supplementary materials.

### Claudin Expression

**Figure 3.**
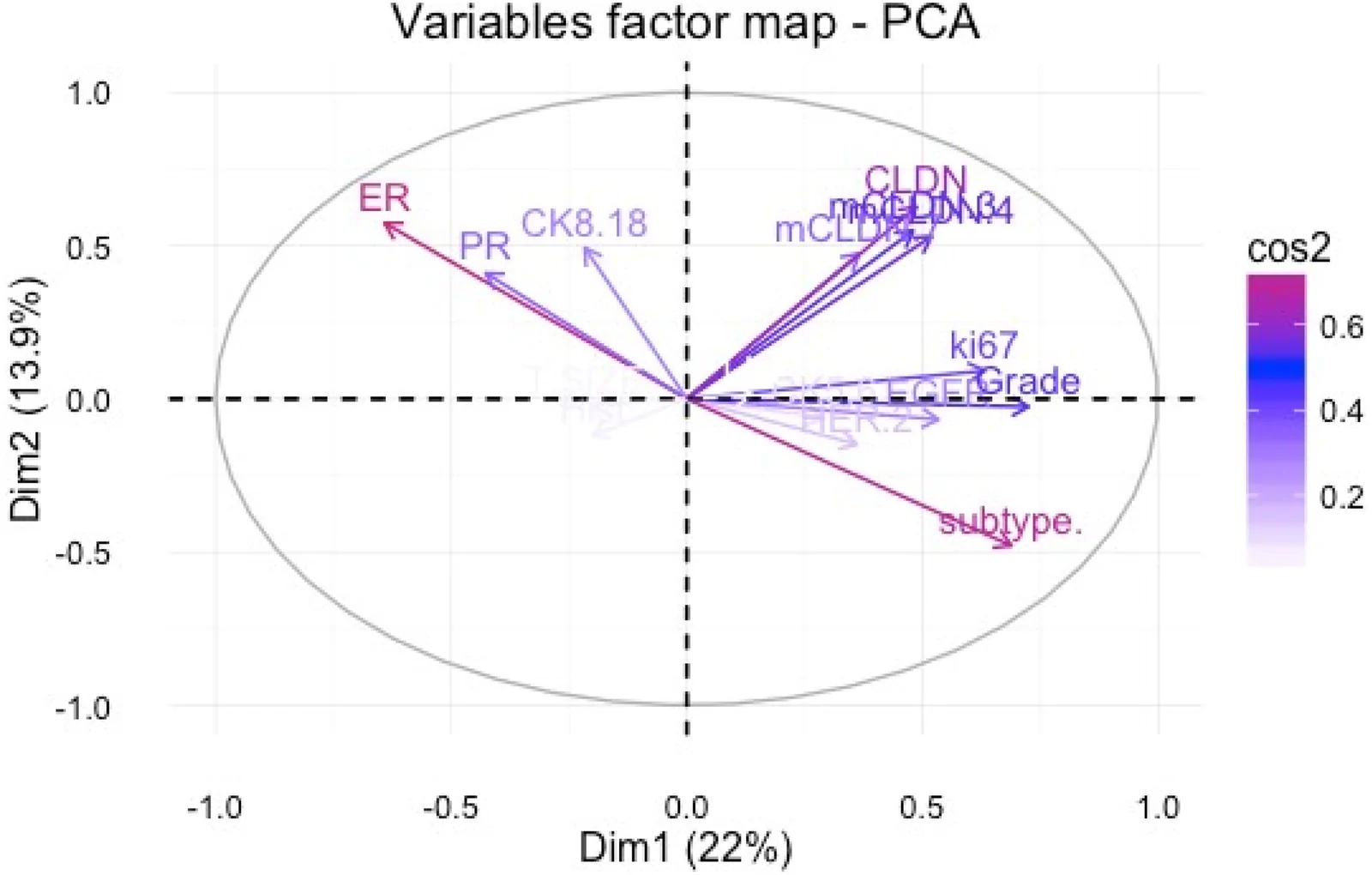
PCA plot showing relationship between claudin membrane proteins and other biomarkers

In the PCA plot claudin-3, claudin-4 and claudin-7 clustered closer to the EGFR and CK5/6 i.e. closer to the TNBC group Figure 3. Signifying that claudin proteins are typical related to independent variables such as proliferation index Ki67 and Grade.

Membranous claudin expression (mCLDN3, 4,7 and overall claudin expression (CLDN) clustered between predictors of aggression (Ki67, EGFR, Grade, HER2 and Molecular subtypes). CK8/18 clustered with good prognostic markers such as ER, PR.

### Claudin Expression Patterns

**Figure 4.**
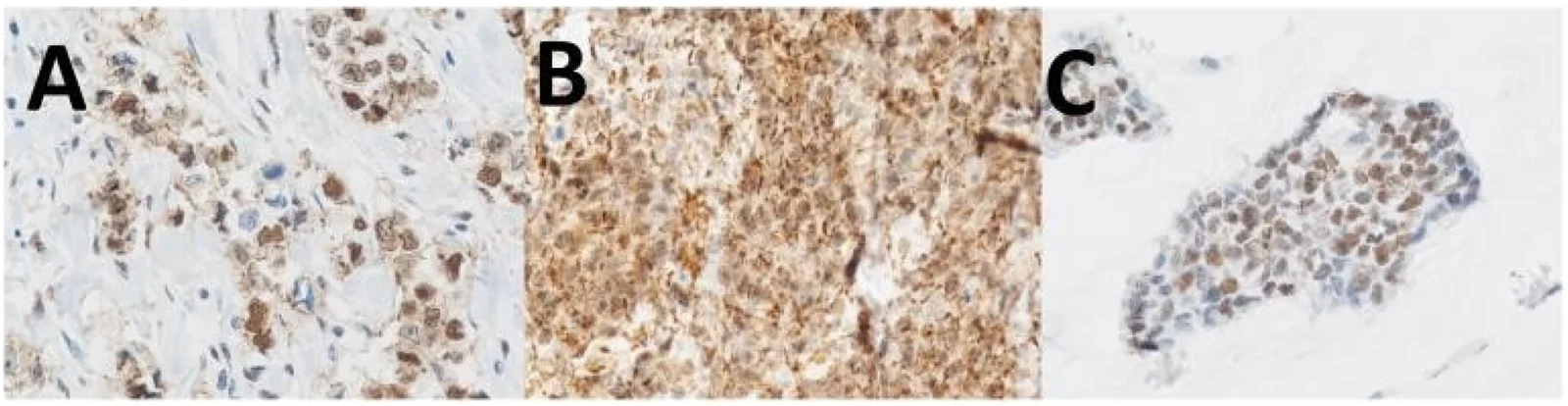
Claudin-3 Expression (A= claudin-3 low, B= claudin 3-high and C =claudin-3 nuclei staining)

Claudin-3 was expressed on the membrane, cytoplasm and to some extent in the nucleus of cancer cells Figure 4. A combination of membrane staining and cytoplasmic expression of claudin-3 was observed in 54% (67/125) cases and 3% (4/125) of these cases were a combination of weak membrane staining and strong cytoplasmic expression. Fifty-eight of tumours were negative or weak positive for claudin-3. Three-quarters (44/58) of tumours which were claudin-3 low showed cytoplasmic, cytoplasmic and nuclear, or nuclear expression [17% (10/58), 12% (7/58) and 47% (27/58) respectively], 12% (7/58) showed a combination of weak cytoplasmic and weak membrane expression. The remaining 12% (7/58) were negative.

**Figure 5.**
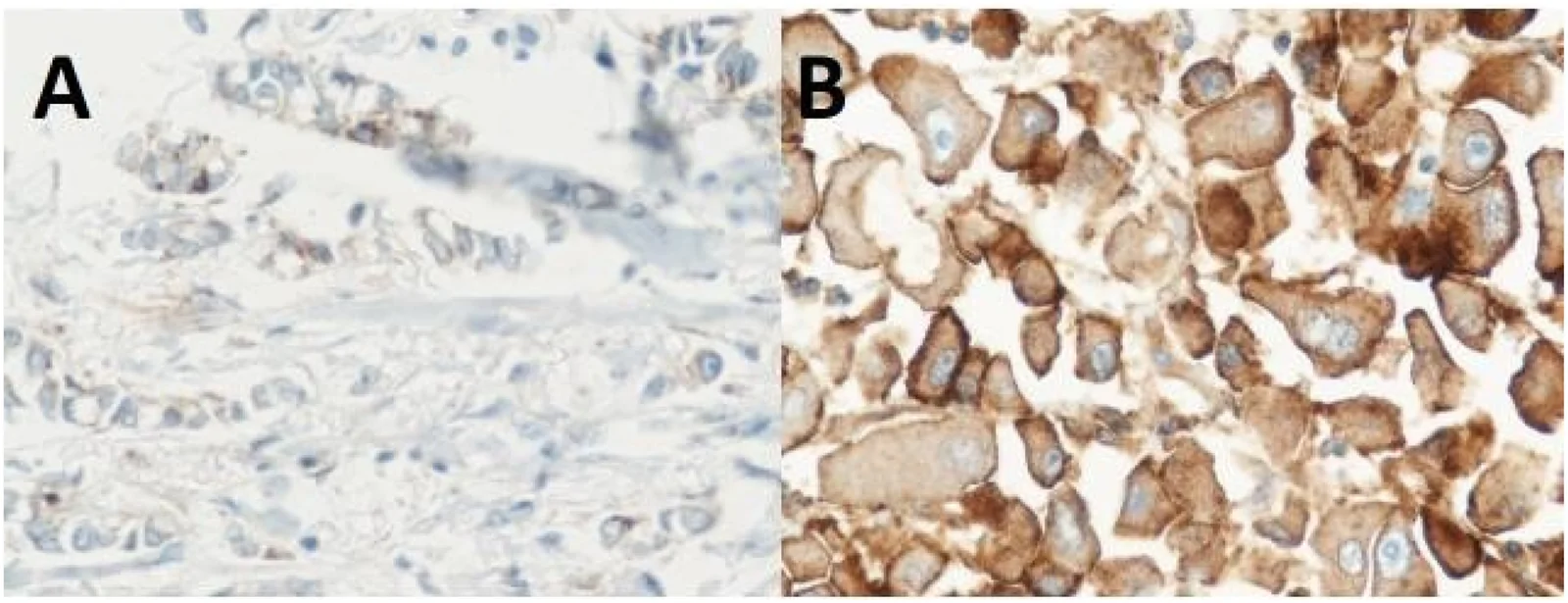
Claudin-4 expression (A, IRS Score =3 and B IRS Score=12)

Claudin-4 was expressed moderately to strong in 53% (66/125) of tumours and of these 55/66 (83%) showed membrane positivity only while the 17% (11/66) showed membrane staining in combination with cytoplasmic staining Figure 5. Out of the remaining 59/125 (47%) that were claudin-4 negative/low, 10% (6/59) showed a combination of weak cytoplasmic and membrane expression, 31% (18/59) cases showed a combination weak cytoplasmic and nuclei expression; with (11/18) showing weak cytoplasmic staining, and (7/18) showing nucleic staining only)). Fifty-nine percent (59%) (35/59) were completely negative.

**Figure 6.**
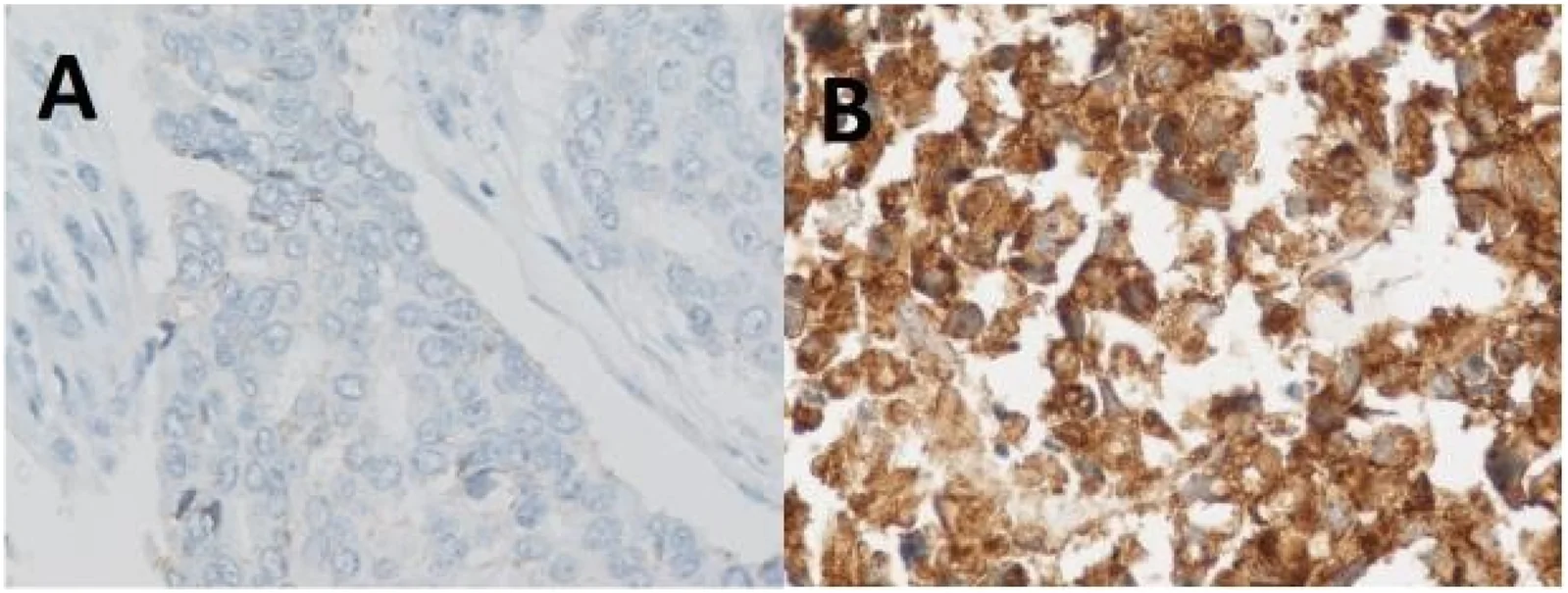
Claudin-7 Expression IRS Scores (A IRS Score=0, B, IRS Score=12) Claudins Correlation with breast cancer molecular subtypes

Claudin-7 was moderate to strong in 47% (59/125) and of these 80% (47/59) showed membrane positivity only while the 20% (12/57) showed strong membrane staining in combination with cytoplasmic staining Figure 6. Of the 53% (66/125) with negative/weak expression; 18% (12/66) showed a combination of cytoplasmic and weak membrane expression, 9% (6/66) showed cytoplasmic staining only and 73% (48/66) were completely negative.

Claudin-3 and Claudin-4 expressions significantly correlated with molecular subtypes (p=0.001) only, Table 4. The majority of Claudin-3 low and claudin 4-low tumours were Luminal A, followed by TNBC.

**Table 4.** Molecular Subtypes Vs Claudin Expression

​	Total n (%)	Molecular subtypes					P Value
		Luminal A n (%)	Luminal B n (%)	Luminal B HER2 n (%)	HER2 enriched n (%)	TNBC n (%)	
**Claudin-3**	​	​	​	​	​	​	**0.001**
Low	58 (46)	29 (50)	2 (3)	5 (9)	6 (10)	16 (28)	​
High	67 (54)	15 (22)	21 (31)	4 (6)	11 (16)	16 (24)	​
**Claudin-4**	​	​	​	​	​	​	0.001
Low	59 (47)	29 (49)	9 (15)	1 (2)	8 (14)	12 (20)	​
High	66 (53)	15 (23)	14 (21)	8 (12)	9 (14)	20 (30)	​
**Claudin-7**	​	​	​	​	​	​	0.408
Low	66 (53)	24 (36)	11 (17)	4 (6)	11 (17)	16 (24)	​
High	59 (47)	20 (34)	12 (20)	5 (9)	6 (10)	16 (27)	​

### Claudins Correlation with biomarkers

Only claudin-7 expression significantly correlated with CK5/6 expression (p=0.029) Table 5. Majority of CK5/6 positive tumours were Claudin-7 high tumours.

**Table 5.** Claudin-7 Expression versus CK5/6 Expression

CK5/6 expression	Claudin-7 low n (%)	Claudin-7 high n (%)	P Value
Positive, n = 16	4 (25)	12 (75)	0.029
Negative, n = 109	62 (57)	47 (43)	​

All Claudins did not correlate with clinicopathological variables Table S2 in supplementary material.

Expression of claudin-3 did not correlate with age (p = 0.576); tumour size (p = 0.711); lymph node involvement (p = 0.828), histological type (p = 0.408) and tumour grade (p=0.138). Claudin-3 low was observed in 42/95 IDC-NOS, 15/28 special histological subtypes and one of the two lobular carcinomas. Claudin-4 had no significant correlation with age (p=0.711), tumour size (p=0.132), lymph node involvement (p=0.661), histological type (p=0.834) and tumour grade (p=0.116). Claudin-7 was not associated with age (p= 0.353); tumour size (p=1.000), lymph node involvement (p=0.383, histological type (p=0.679),) and tumour grade (p=0.501).

Claudin-3 expression did not correlate with expression: of EGFR (p=0.064), CK8/18 (p=0.618) and CK5/6 (p=0.826) Table S2. Claudin-4 expressions did not correlate with: EGFR (p=0.185), CK8/18 (p= 0.193) and CK5/6 (p=1.000) Table S2. Likewise, Claudin-7 expression was not related to molecular subtype (p=0.408), EGFR (p=0.258) and CK8/18 expression (p=0.137) Table S2.

### Claudin-low Classification

Claudin-low describes a breast tumour with negative or low expression of all three principal claudins: claudin-3, claudin-4 and claudin-7 (≤25% staining, IRS Scores 0-3). Claudin-high describes a breast tumour with an expression >25% in at least one or more of either claudin-3, claudin-4 and claudin-7. Twenty-seven tumours were claudin-low and 98 were claudin-high. The distribution of claudin-low tumours within breast molecular subtypes were as follows: 52% (14/27) were luminal A, 29% (8/27) TNBC, 11% (3/27) HER2OE, while Luminal B and Luminal B HER2 subtypes accounted for 4% (1/27) each, Table 6.

**Table 6.** Distribution of Claudin-Low Amongst Molecular Subtypes

Molecular subtype	Frequency n (%)	Claudin-low n (%)	p-value
Luminal A	44 (35)	14 (52)	p=0.312
Luminal B	23 (18)	1 (4)	
Luminal B HER2	9 (7)	1 (4)	
TNBC	32 (26)	8 (29)	
HER2 OE	17 (14)	3 (11)	
TOTAL	125 (100 %)	27 (100)	

### Claudin-low subtype

Claudin-3, claudin-4 and claudin-7 were downregulated with an average IHC IRS score of (1.2/12), (1.5/12) and (1.3/12), respectively. Frequency of the claudin-low subtype in the Botswana female breast cancer cohort was 22% (27/125). The median age was 57 years, and the age range was 35 to 87 years. Sixteen (16/27) IDC-NOS and 11 were histological special types. There were 3/27 grade 3 tumours, 8/27 grade 2 tumours, 9/27 tumours grade 1 tumours, and 7/27 mucinous carcinomas.

### Claudin-Low Correlation With Clinicopathological Variables and Biomarkers

Claudin-low breast cancer subtype significant associated with histological type (p=0.002), tumour size (p= 0.013) and lymph node involvement (p< 0,001). Most claudin-low tumours were infiltrating Ductal Carcinoma of no specific features IDC-NOS Table 7 above. Over two thirds (68%) of claudin-low tumours were larger than 5 cm. IDC-NOS accounted for 59% (16/27) of claudin-low tumours and 41% (11/27) were histological special subtypes.

**Table 7.** Claudin-Low versus Histological Type

Variable	Claudin- high n (%)	Claudin-low n (%)	P value
**Histology type**	​	​	0.002
IDC-NOS n=93	79 (81%)	16 (59%)	​
Special types n=28	17 (17%)	11 (41%)	​
Lobular ca n=2	2 (2.0%)	0 (0%)	​

Variable	Claudin- high n (%)	Claudin-low n (%)	P value
* **Tumour Size**	​	​	0.013
<5 cm (AJCC *Tmi to T1c,* AJCC *T2*) n=63	55 (61)	8 (32)	​
>5 cm (AJCC *T3 & T4*) n=52	35 (39)	17 (68)	​

*Upon excluding 10 tumours with missing tumour size.

Overall claudin status did not correlate with clinicopathological variables; tumour grade (p=0.363, age (p=0.656), lymph node involvement (p=0.791), and biomarkers for: CK5/6 (p=0.521), CK8/18 (p=0.175) and EGFR (p=0.122) Table S3 in Supplementary materials.

## Discussion

CK8/18 has been explored in breast cancer studies conducted in the West and North Africa^40,41^ either as CK18 or CK8/18 and our study is the first study in the Southern Africa. We employed H-scores to quantify and to establish cut-off values for its positivity as in previous studies.^40-42^

In the Botswana cohort, overall expression of CK8/18 as determined by the H-score was 90% and the mean expression was 125 ± 84 (median 115, range 0-300). The cut-off values for CK8/18 positivity were set at one-third of total score i.e. 100 for H-score.^40,43^ Sixty-four percent, (64%) of our cohort tested positive for CK8/18 and this was similar in both age groups Table 3. This expression is consistent with that observed in a Nigerian study.^42^ In an Egyptian study, all cases were positive.^41^ Given that in this cohort (Table 2) had an ER positivity of 57% and high prevalence of TBNC and tumour heterogeneity,^34^ prevalence of CK8/18 is less than that of Egyptian cohort as it is gradually reduced in tumours with such traits. There was a strong association between tumour grade and CK8/18 and loss of its expression has been associated with poorly differentiated breast carcinoma.^22,43,44^ This finding is consistent with findings from other studies.^26,29,41^ The association of CK8/18 with grade could be attributed to the function of CK8/18, it is an intermediate protein that is involved in cytoskeleton and maintenance of cellular integrity.^24,45^

CK8/18 expression had a significant association with breast cancer molecular subtype Table 3. CK8/18 has been associated with good prognosis as its expression is associated with luminal subtypes (tumours which are ER positive).^26,46^ CK8/18 is involved in the anchoring of the ERα and maintenance of the nucleus integrity. Hence Most ER positive tumours expresses CK8/18.^24,26^ This result is consistent with findings from Egypt and Nigeria.^41,42^

Functional studies have proved that addition of CK8/18 to a panel of prognostic markers can help identify low ER positivity tumours which could benefit from second line breast cancer therapy drug fluvestrant.^24,25^ And in a recent clinical study a combination of culmerciclib and fulvestrant significantly improved prognostic progression-free survival gains to HR positive HER2 negative patients.^47^ Uptake of this drug through the CK8/18 signalling could help in the treatment of patients who express low ER but have a high expression CK8/18, this phenomenon has been attributed to loss of ER.^25,48,49^ CK8/18 interacts with ERα in normal physiology to facilitate its degradation via the ubiquitin-proteosome pathway.^24^

CK8/18 is signature of normal luminally well differentiated epithelia cells and luminal breast cancer are thought to arise from these cells.^50^ Normal cells express low Ki67 and high CK8/18 while in cancer there is loss of CK8/18^25,102^. CK8/18 was positively associated with the molecular classification table 3, luminal A had the highest mean expression of CK8/18. Membrane staining CK8/18 expression was associated with luminal subtypes, while decreased expression was associated with TNBC and HER2 enriched subtypes. This observation is consistent with studies by Aiad et al, (2014) and Yadav et al, (2016).^41,51^

CK8/18 is high in luminal A tumours (have a proliferation index Ki67 score ≤14.0% and gradual decreases in subtypes with Ki67 index > 14% Table 3. CK8/18 is involved in cell proliferation and apoptosis.^52^ CK8/18 binds to the cytoplasmic domain of tumour necrosis factor 2, (TNFR2) and influences the TNF-induced c-jun-NH2-kinase (JNK) signalling to effect cellular proliferation, differentiation, survival and death in cell as part of its normal cellular functions.^53,54^ In rapidly dividing tumour cells, there is down-regulation of CK18/8 due to destabilization and disruption of cytokeratin network in the cell. CK8/18 is recruited and phosphorylated by p38 during the mitosis phase.^22,45^

CK8/18 is required in the maintenance of cell structures, support and hold several proteins in position including oestrogen receptor, ER. It is also involved in apoptosis as it facilitates autophagy,^55^ hence the differential loss of CK8/18 in TNBC than in well differentiated luminal tumours. CK8/18 protects epithelial cell from metabolic stress^56^ and in cell signalling during apoptosis. Thus, an intact signalling keratin network is associated with less aggressive tumours.^57^ CK8/18 is involved in the p38MAPK signalling pathway, the p38 phosphorylate both CK8 and CK18 at Serine residue 73 and 52 respectively in response to cellular stress and during mitosis^58,59^

CK8/18 gradual reduces in luminal B and HER2 enriched tumours (Ki67 index >14%) and since CK8/18 is involved in the process mitosis. CK8/18 and ER are directly proportional to each other, and molecular subtypes are a derivative of ER expression.

The HER2 enriched group was characterized by low expression of CK8/18 (average H score 101). In this cohort the Notch signalling pathway could be the most potent HER2 signalling due to elevation of both EGFR (HER1) and HER2.^34,60^ Dimerization of HER2/HER2 or HER1/HER1 and HER1/HER2 has been associated with EMT through the Notch signalling.^61^ This signalling is accompanied by relocation of EGFR to the nucleus where it affects transcription of other genes which further participates in Notch signalling like SLUG which represses CK8/18 resulting in metastasis.^62^

CK8/18 did not correlate with remaining clinicopathological variable and biomarkers Table S1. There was no significant difference between age as observed previously by Dialla et al (2015).^63^

### Claudin-Low Molecular Breast Cancer Subtype

The role of claudin proteins remains unexplored in SSA breast cancer studies and the current study is the first study in Africa. There is therefore a clarion call to investigate disparities brought about by breast cancer biological difference between women of African descent and those of their Caucasian counterparts.^64,65^ Claudin-low is defined as tumours with concurrent IRS scores less than 3/12 for claudin3,4 and 7 (intensity 0-3, proportion 0-4). Claudin expression was explored in this population because most patients presented with large tumours with immune infiltrating lymphocytes, a phenomenon first described by Herschkowitz et al (2007).^15^ The average largest dimension of tumour was 5.7 cm in women aged ≤ 50 years in this cohort. Hoevell et al, (2004) found that claudin repression encourages apoptosis. Claudin -3 and claudin-4 serve as constrainers of tumour growth^66,67^ and participate in cell signaling through MAPK and other signaling pathways.^14^ Hence it was hypothesized that large breast tumours in women of African descent could be attributed to weak tight junctions which permit passage of growth factors and fail to constrain expanding tumours.^66-68^ According to Harrel et al, (2014) Claudin-low tumours promote tumour vascular permeability and metastasis.^69^ Therefore, investigations into the role of claudins was done across all molecular subtypes as there is evidence that women of African descent present with large, clinically advanced stage tumours across all molecular subtypes.^5-11^ Furthermore, there is tumour heterogeneity,^34^ which is associated with under differentiation, a feature associated with claudin-low tumours.^70-72^

In our cohort claudins low expressing tumours were found to cut across all molecular subtypes Table 6. Previously claudin-low expressing tumours were associated with TNBC molecular subtype but in our cohort claudin-low did not correlate with molecular subtypes Table 6. This has been also emphasised in a review by Fougner et al 2020, which defined claudin-low breast cancer as an independent breast cancer phenotype that cut across all molecular subtypes.^23^

These tumours were characterized by molecular heterogeneity and 44% of them expressed EGFR, the majority with nuclear positivity. Nuclear translocation of EGFR has been associated with therapy (Gefitinib) resistance in breast cancer.^73,74^ AKT phosphorylation facilitated nuclear translocation of EGFR which enhances transcription of breast cancer resistant protein.^73^

Claudin-low tumours had an average proliferation index of 12%, decreased CK8/18 expression and majority of them were IDC NOS (16/27).

Claudin-low tumours have been reported in the ER positive and HER2 enriched subtypes.^72,75-78^ Szasz et al, (2011) hypothesized that there might be claudin-low tumours within the luminal subtypes and HER2 enriched tumours depicted as outliers in the original gene expression microarray that was employed to derive intrinsic breast cancer molecular subtypes.^32,76^

Claudin-3 and Claudin-4 significantly corelated with molecular subtypes Table 4, most of claudin-3 low and claudin-4 low tumours were luminal (63% & 64% respectively) and TNBC (28% and 20% respectively). This finding is consistent with a finding that claudins expression vary with specific molecular breast cancer subtypes^79^ and tumour grade.^17^ Claudin 3 and claudin 4 have been found to have significant prognostic value in luminal and TNBC.^80^ Thus, tumours with downregulated claudin-3 and claudin-4 were confined to luminal A subtypes while those with upregulated claudin-3 and claudin-4 were confined to fast proliferating tumours in TNBC and luminal B subtypes and these findings are in agreement with other findings.^68,75,81,82^

Claudin-low tumours are generally regarded as the most primitive form in terms of differentiation, but in this cohort, 15 tumours were ER positive and 6/15 were mucinous tumours which has a natural disrupted membrane protein.^83^ Of the 5/15 ER positive, 2 expressed nuclear EGFR and 3 had a low ER Allred score, (although correlation between Claudin-low profile and EGFR was not statistically significant) this mixed phenotype could be attributed to the natural heterogeneity of breast tumours.^84,85^ There is also a possibility that they are an emerging residual phenotype following treatment which could have ablated the tumour cells with luminal phenotype.^86-88^

The other plausible reason could be that luminal A tumour cells are undergoing EMT^89^ through Notch signaling.^62^ Low expression of claudin-3, 4 and 7 in ER positive and HER2 enriched tumours could be attributed to EMT as most of these tumours expressed cytoplasmic and nuclear EGFR.^14,62^ Claudin-low subtype tumours have better prognosis in the short term as they are slow proliferating tumours but as time passes by their cumulative build-up of detrimental features (Cancer stem cells and EMT) becomes the difference.^72,75,76,90^ A larger study is therefore proposed to assess the impact of claudin-low on women of African descent. The current study is larger than a previous study which has a cohort 24 African American in its cohort but was confined to IDC grade 3 tumours only.^77^

The ER negative, claudin-low group had decreased CK8/18 expression; 8/11 tumours expressed EGFR, and 5/11 of these tumours expressed nuclear EGFR which has been associated with radiotherapy resistance^91^ and had decreased CK8/18. Of the 11 ER negative claudin-low expressing tumours; 3 were HER2 enriched, and 8/11 tumours were TNBC. These tumours fit the conventional description of claudin-low tumours.^15,17,92^

Claudin-7 significantly corelated with CK5/6 Table 5, this finding is consistent with a finding previous studies^77,93^ thus aligning claudin-7 low expressing phenotype to the basal subtype.

Claudin-low significantly correlated with histological subtypes, tumour size a (upon adjusting for those missing data) Table 7, most claudin-low tumours are IDC-NOS and is consistent with studies by Park et al (2007) and Weigelt, Geyer and Reis-Filho (2010).^30,94^ Most claudin-low tumours were bigger than 5 cm a trait observed by Dias et al (2017) therefore their absence/down regulated claudin proteins results in large tumours.^13,90,92^

All claudins proteins (claudin-3, 4 &7) did not corelate with clinicopathological variables (Table S2) and likewise claudin-low profile did not correlate with biomarkers and the rest of clinicopathological variables (Table S3 in supplementary materials), which could be due to the small cohort when compared to other studies.^80,95,96^

### Limitations

A small sample size emanating from a small population size of Botswana and also from suboptimal tissue may have limited the statistical power of the study for some variables. A study involving more centres in the country and neighbouring countries is being considered.

The study utilised IHC for classification, although IHC has limitation associated with protein fixation, many other studied have used it and achieved results with IRS positivity cut-off set at 33% (3/9), our study used an expanded IRS for claudin expression (expanded from 0-9 to 0-12).^97^ Our cut off was set at a stringent cut off of 25% (3/12) in an expanded IRS scoring system.^68,77,90^ Furthermore, a study on gene expression for claudin 3, 4 and 7 on this Botswana cohort is planned.

## Conclusions and Recommendations

CK8/18 is a promising prognostic marker and could be used to identify patients with poor tamoxifen response who could benefit from the second line drug Fulvestrant.

The findings of this research will be communicated to the Botswana Ministry of Health policy makers and the National standing committee on drugs to explore the feasibility of employing Fluvestrant for patients with low ER expression, currently its availed on special request basis.

Large tumours observed in this cohort are attributed to weakened tight junction proteins, i.e. claudin-low tumours as they were spread across all molecular subtypes. Identification of Claudin-low from conventional breast cancer molecular subtypes could enhance diagnostic and prognostic value for patient diagnosed with this phenotype as they respond differently to conventional therapy. Currently immunotherapy employing anti-claudin 3^98^ and claudin 4^99^ are in development, raising hope of a claudin specific molecular directed therapy^100^ synonymous with Herceptin employed for HER2 enriched molecular breast cancer subtype. **5749**.

## Supplemental Material

Supplemental Material - CK8/18 and Claudin Profile of Female Breast Cancers from Botswana Supplemental Material for CK8/18 and Claudin Profile of Female Breast Cancers from Botswana by Andrew Khulekani Ndlovu, Ishmael Kasvosve, Richard Naidoo, Dhiren Govender in Breast Cancer: Basic and Clinical Research.

## Data Availability

All data used/analysed during the study is available from the corresponding author on reasonable request.*

## Appendix

Abbreviations
NCD: Non-communicable diseases
LMIC: Low- and middle-income countries
GLOBOCAN: Global cancer statistics
IARC: International Agency for Research on Cancer
GNI: Gross national income
SSA: Sub-Saharan Africa
ASR: Age Standardized rate
UMIC: Upper Middle-Income country
BNCR: Botswana National Cancer Registry
SAMBER: Statistical Analysis and Methods in Biomedical Research
DCIS: Ductal carcinoma in situ carcinoma
LCIS: Lobular carcinoma in situ
IDC-NOS: Invasive Ductal Carcinoma, not otherwise Specified
H&E: Haematoxylin and Eosin
IHC: Immunohistochemistry
ER: Oestrogen receptor
PR: Progesterone receptor
HER2/neu: Human Epidermal Growth Factor Receptor 2
CK8/18: Cytokeratin 8/18
CLDN: Claudin
PMH: Princess Marina Hospital
NRH: Nyangabgwe Referral Hospital
UCT: University of Cape town
UB: University of Botswana.
