# Supplementary material for: CK8/18 and Claudin Profile of Female Breast Cancers from Botswana
Source: Breast Cancer (Auckl). 2026 Aug 27;20:11782234261471677. doi: 10.1177/11782234261471677 (PMC13525293; doi:10.1177/11782234261471677)
Supplement: Supplementary file 1 [file sj-pdf-1-bcb-10.1177_11782234261471677.pdf]

Supplementary Materials

Table S1: Clinicopathological variables and biomarkers that had no significant correlation with of CK8/18

Variable	CK8/18		
	**Positive n= (%)**	**Negative n= (%)**	**p-value**
**Age**			
<50 years, n=45	30 (67)	15 (33)	0.847
>50 years, n=80	55 (69)	25 (31)	
**Tumour Size**			
Tumour Size <5cm, n=63	46 (73)	17 (27)	0.326
Tumour Size >5cm, n=52	34 (65)	18 (35)	
Unknown, n=10	5 (50)	5 (50)	
**Lymph Node Involvement (LNI)**			
Positive LNI, n=83	55 (66)	28 (34)	0.269
Negative LNI, n=28	20 (71)	8 (29)	
Unknown, n=14	10 (71)	4 (29)	
**Histology type**			
IDC-NOS n=93	68 (73)	27 (27)	0.165
Special types n=28	15 (53)	13 (47)	
Lobular ca n=2	2 (100)	0 (0)	
**Biomarkers**			
CK5/6 positive, n=16	14 (88)	2 (12)	0.785
CK5/6 negative, n=109	84 (77)	25 (230	
EGFR positive, n=42	23 (55)	19 (45)	0.075
EFGR negative, n=83	62 (75)	21 (25)	

Table S2: Expression of Cludin-3, Claudin-4 and Claudin-7 versus clinicopathological variables and biomarkers

Variable	Claudin-3			Claudin-4			Claudin-7		
	pos	neg	p-value	pos	neg	p-value	pos	neg	p-value
Age									
Age <50	26	19	p = 0.576	25	20	0.711	24	21	0.353
Age>50	41	39		39	41		35	45	
Tumour Size <5cm	35	28	p = 0.711	39	24	0.132	28	35	1.000
Tumour Size >5cm	27	25		25	27		24	28	
Unknown	5	5		3	7		7	3	
**Lymph Node Involvement (LNI)**									
Positive LNI	45	38	0.828	46	37	0.661	40	43	0.383
Negative LNI	13	15		14	14		12	16	
Unknown	9	5		10	4		7	7	
**Histology type**									
IDC-NOS	51	42	0.408	50	43	0.834	46	47	0.679
Special types	15	15		15	15		13	17	
Lobular	1	1		1	1		0	2	
**Tumour grade**									
Grade 1	17	21	0.138	16	22	0.116	17	21	0.501
Grade 2	29	21		32	18		24	26	
Grade 3	20	9		17	12		17	12	
Mucinous	1	7		1	7		1	7	
Biomarkers									
CK818 Positive	44	36	0.618	46	34	0.193	42	38	0.137
CK8/18 negative	23	22		20	25		17	28	
**CK5/6 positive**	**7**	**60**	**0.826**	**8**	**58**	**1.000**	**12**	**47**	**0.029**
**CK5/6 negative**	**9**	**49**		**8**	**51**		**4**	**62**	
EGFR positive	26	41	0.064	26	40	0.185	23	36	0.258
EFGR negative	16	42		16	43		19	47	

Table S3: Claudin-low versus clinicopathological variables and biomarkers

Variable	Claudin-Status		
	CLDN-High n=98 (%)	CLDN-Low n=27 (%)	p-value
Age			
<50 years	37	8	0.656
>50 years	61	19	
**Lymph Node Involvement (LNI)**			
Positive LNI	69	14	0,3087
Negative LNI	23	5	
Unknown	6	8	
**Histology type**			
**IDC-NOS n=93**	**79 (81%)**	**16 (59%)**	**0.002**
**Special types n=28**	**17 (17%)**	**11 (41%)**	
**Lobular ca n=2**	**2 (2.0%)**	**0 (0%)**	
Tumour grade			
Grade 1	29	9	0.363
Grade 2	42	8	
Grade 3	26	3	
Mucinous	1	7	
Biomarkers			
CK818 Positive	69	16	0.175
CK8/18 negative	29	11	
CK5/6 positive	14	2	0.521
CK5/6 negative	84	25	
EGFR positive	36	6	0.122
EFGR negative	62	21	
